# Safety, tolerability, clinical, and joint structural outcomes of a single intra-articular injection of allogeneic mesenchymal precursor cells in patients following anterior cruciate ligament reconstruction: a controlled double-blind randomised trial

**DOI:** 10.1186/s13075-017-1391-0

**Published:** 2017-08-02

**Authors:** Yuanyuan Wang, Andrew Shimmin, Peter Ghosh, Paul Marks, James Linklater, David Connell, Stephen Hall, Donna Skerrett, Silviu Itescu, Flavia M. Cicuttini

**Affiliations:** 1 Department of Epidemiology and Preventive Medicine, School of Public Health and Preventive Medicine, Monash University, Alfred Hospital, Melbourne, VIC 3004 Australia; 2 Melbourne Orthopaedic Group, 33 The Avenue, Windsor, VIC 3181 Australia; 3 Mesoblast Ltd., Level 38, 55 Collins Street, Melbourne, VIC 3000 Australia; 4 Imaging Associates Box Hill, Box Hill, VIC 3128 Australia; 5 Castlereagh Imaging, 60 Pacific Highway, St Leonards, NSW 2065 Australia; 6 Imaging @ Olympic Park, AAMI Park, 60 Olympic Boulevard, Melbourne, VIC 3000 Australia; 7 Emeritus Research, 291 Wattletree Road, Malvern East, VIC 3145 Australia

**Keywords:** Mesenchymal precursor cells, Anterior cruciate ligament reconstruction, Pain, Function, Cartilage, Subchondral bone, Post-traumatic osteoarthritis

## Abstract

**Background:**

Few clinical trials have investigated the safety and efficacy of mesenchymal stem cells for the management of post-traumatic osteoarthritis. The objectives of this pilot study were to determine the safety and tolerability and to explore the efficacy of a single intra-articular injection of allogeneic human mesenchymal precursor cells (MPCs) to improve clinical symptoms and retard joint structural deterioration over 24 months in patients following anterior cruciate ligament (ACL) reconstruction.

**Methods:**

In this phase Ib/IIa, double-blind, active comparator clinical study, 17 patients aged 18–40 years with unilateral ACL reconstruction were randomized (2:1) to receive either a single intra-articular injection of 75 million allogeneic MPCs suspended in hyaluronan (HA) (MPC + HA group) (*n* = 11) or HA alone (*n* = 6). Patients were monitored for adverse events. Immunogenicity was evaluated by anti-HLA panel reactive antibodies (PRA) against class I and II HLAs determined by flow cytometry. Pain, function, and quality of life were assessed using the Knee Injury and Osteoarthritis Outcome Score (KOOS) and SF-36v2 scores. Joint space width was measured from radiographs, and tibial cartilage volume and bone area assessed from magnetic resonance imaging (MRI).

**Results:**

Moderate arthralgia and swelling within 24 h following injection that subsided were observed in 4 out of 11 in the MPC + HA group and 0 out of 6 HA controls. No cell-related serious adverse effects were observed. Increases in class I PRA >10% were observed at week 4 in the MPC + HA group that decreased to baseline levels by week 104. Compared with the HA group, MPC + HA-treated patients showed greater improvements in KOOS pain, symptom, activities of daily living, and SF-36 bodily pain scores (*p* < 0.05). The MPC + HA group had reduced medial and lateral tibiofemoral joint space narrowing (*p* < 0.05), less tibial bone expansion (0.5% vs 4.0% over 26 weeks, *p* = 0.02), and a trend towards reduced tibial cartilage volume loss (0.7% vs –4.0% over 26 weeks, *p* = 0.10) than the HA controls.

**Conclusions:**

Intra-articular administration of a single allogeneic MPC injection following ACL reconstruction was safe, well tolerated, and may improve symptoms and structural outcomes. These findings suggest that MPCs warrant further investigations as they may modulate some of the pathological processes responsible for the development of post-traumatic osteoarthritis following ACL reconstruction.

**Trial registration:**

ClinicalTrials.gov (NCT01088191) registration date: March 11, 2010﻿

**Electronic supplementary material:**

The online version of this article (doi:10.1186/s13075-017-1391-0) contains supplementary material, which is available to authorized users.

## Background

Osteoarthritis (OA) is a widespread debilitating chronic condition that causes joint pain, functional disability, and impaired quality of life. It is a disease with a multifactorial aetiology, with obesity, ageing, occupational, hormonal, and genetic factors considered to be important contributors to its onset and progression [1]. Joint injury is also a well-established risk factor for the development of OA [2]. For approximately 12% of the OA population the condition is considered to arise secondary to a traumatic injury [3], leading to the classification of this group of patients as presenting with post-traumatic OA. Post-traumatic OA affects 5.6 million individuals in the United States with an annual direct medical cost of approximately $3 billion [3]. Anterior cruciate ligament (ACL) rupture is a relatively common injury, particularly during sporting activities. Individuals who sustained an ACL rupture account for an estimated 25% of the overall knee OA population [4, 5]. Approximately 80% of patients with traumatic rupture of the ACL show evidence of radiographic OA within 12–14 years following the injury, despite surgical repair [6, 7]. Moreover, there is no evidence that ACL reconstruction improves knee joint structural outcomes and the onset of OA [8].

Currently used modalities for the treatment of post-traumatic OA address the symptoms that arise subsequent to the initiating injury but, apart from their analgesic and anti-inflammatory activities [9, 10], offer limited value in restoring the joint structural changes induced by the initial traumatic event or diminishing the progression to symptomatic OA. Adult mesenchymal stem cells (MSC) are an abundant source of self-renewing, multipotent, undifferentiated cells that can be readily isolated from bone marrow, adipose tissue, muscle, or synovium and then readily culture expanded without undergoing differentiation [11]. The ability of these cells to differentiate into bone, cartilage, adipose, tendon, and other cells of the mesenchymal lineage under appropriate stimuli suggests the potential for the regeneration and repair of injured joint tissues in the clinic [11, 12]. Moreover, MSC exhibit trophic, anti-inflammatory, and immunomodulatory activities that have been shown to attenuate the mediators of joint tissue destruction as well as promoting their repair [11–14]. As a consequence, MSC have been investigated for their therapeutic potential for the treatment of established OA and other arthropathies [13, 15–19]. Positive outcomes from these studies have been reported in terms of safety and the modulation of joint tissue breakdown as well as improving joint pain and function, but for the most part the MSC used were autologous, were derived from a variety of sources, and often administered as multiple doses [13, 15–19]. We are aware of only one randomised double-blind, placebo-controlled clinical trial that investigated the safety and efficacy of allogeneic MSC for the management of post-traumatic OA [19]. In this 24-month trial a single intra-articular injection of allogeneic bone marrow-derived MSC, suspended in hyaluronan (HA), was administered within 7 to 10 days of surgery to 55 patients who has been subjected to partial meniscectomy [19]. Although only 23% of the patients who received the lower intra-articular dose of 50 million MSC + HA showed greater than 15% increase in meniscus volume post-treatment, none of the HA-injected controls achieved this level of meniscal regeneration [19]. Moreover, over the course of the study, no safety concerns were reported, and significant improvements in OA symptoms and decreased progression of magnetic resonance imaging (MRI) indices of disease progression were observed relative to the HA-alone injected group [19].

Stromal tissue within the perivascular niche of the bone marrow contains a heterogeneous population of MSC that may be isolated by ficoll gradient centrifugation, followed by adherence in plastic culture flasks [20]. MSC preparations derived from adult human bone marrow by this means contain low abundance of colony-forming units-fibroblast (CFU-F) and are contaminated with mature stromal cells, non-mesenchymal cell types, and a small population of immature mesenchymal precursor cells (MPCs) [20, 21]. However, MPCs can be separated from the MSC and other stromal elements by magnetic-activated cell sorting using specific monoclonal antibodies that bind to antigens expressed on the surface of the MPCs [22, 23]. Examples of these antibodies include: STRO-1, STRO-3, STRO-4 (HSP-90b), VCAM-1 (CD106), and CD146 [23–26]. The MPCs isolated using these antibodies for immuno-selection lack the phenotypic characteristics of mature stromal elements and include the majority of the CFU-F and plasticity present in the plastic-adherent bone marrow MSC population [23, 25, 27]. Previous studies utilising immune-selected STRO-1^+^ or STRO-3^+^ MPCs demonstrated that they exhibited superior clonogenicity, proliferative efficiency, and maintained their immunophenotype following culture expansion compared to plastic-adherent MSC isolated from the same bone marrow aspirates [27–29].

Our previous preclinical studies using ovine models of post-traumatic OA [30] and collagen-induced arthritis [31, 32] have demonstrated the safety and efficacy of allogeneic STRO-3^+^-selected MPCs in preserving synovial joint articular cartilage, downregulating pro-inflammatory cytokine activities, and preserving cartilage and endothelial cell function. These encouraging preclinical findings using allogeneic STRO-3^+^ MPCs provided the rationale for our hypothesis that these cells were safe and might provide long-term clinical and joint structural benefits when administered to patients after ACL reconstruction. Accordingly, the primary aims of the present study were to evaluate the safety and tolerability over 24 months of a single intra-articular injection of 75 million allogeneic MPCs suspended in HA compared to injection of HA alone when administered to patients post-ACL rupture and surgical reconstruction. A secondary objective was to explore the efficacy of these agents in improving clinical outcomes, joint cartilage integrity, and subchondral bone remodelling over 24 months using radiographs and MRI.

## Methods

### Study design, setting, and participants

This study was a single centre, phase Ib/IIa randomised, double-blind, parallel group, active comparator clinical trial conducted in Melbourne, Australia, using criteria compliant with principles of good clinical practice (cGCP) and in accordance with the declaration of Helsinki as mandated by the Therapeutic Goods Administration (TGA) of Australia. The patient group recruited for the study during 2009–2012 consisted of 17 young adults aged 18–40 years who had sustained an initial, unilateral ACL injury and were subjected to an ACL reconstruction within 6 months of the acute injury but had no visual evidence of joint articular cartilage lesions when viewed at the time of surgery. The details of inclusion and exclusion criteria are shown in Table 1. The trial was registered at ClinicalTrials.gov (NCT01088191) prior to recruitment. Ethics approval was obtained from the Cabrini Human Research Ethics Committee. All participants provided written informed consent before their participation.

**Table 1:** Inclusion and exclusion criteria

Inclusion criteria
(1) Males or females aged 18–40 years; (2) Anterior cruciate ligament (ACL) injury requiring reconstruction with bone bruising evident on pre-operative magnetic resonance imaging (MRI) scan at screen or within 6 months of initial ACL injury; (3) Have undergone unilateral ACL reconstruction surgery within 6 months of injury; (4) Clinically stable knee after reconstruction—International Knee Documentation Committee clinical knee examination at time of surgery after reconstruction to be grade normal or nearly normal; (5) Willing and able to undertake a standardized rehabilitation protocol as assessed by surgeon; (6) ACL graft used was autograft hamstring; (7) Willingness to participate in follow-up for 24 months from the time of initial treatment; (8) Ability to understand and willingness to sign consent form; (9) If a female was of childbearing potential, then she must have confirmed negative urine and serum pregnancy test result at screening, and a negative urine pregnancy test prior to the administration of the study treatment, and agree to use a medically reliable method of preventing conception for the first 6 months after injection of the study treatment; (10) Male patients with partners of childbearing potential must be willing to use a medically reliable method of preventing conception for the first 6 months after injection of the study treatment.
Exclusion criteria
(1) Women who are pregnant or breast feeding or planning to become pregnant during the first 6 months after injection of the study treatment; (2) Known sensitivities to bovine (cow), murine (mouse), chicken products, and/or dimethyl sulphoxide; (3) Known allergies to products from birds such as feathers, eggs, or poultry; (4) Previous allergic reaction to hyaluronan (HA); (5) Systemic or local infection at the screen visit or at the time of the study injection; (6) History of any autoimmune disease, such as systemic lupus erythematosus, Addison’s disease, Crohn’s disease, or rheumatoid arthritis; (7) Treatment with immunosuppression therapy within 6 months prior to screening; (8) Chronic (at least 7 consecutive days) of systemic corticosteroids at a dose equivalent to >10 mg/day prednisolone within 14 days prior to screening; (9) Acute or chronic infectious disease, including but not limited to human immunodeficiency virus; (10) Treatment and/or uncompleted follow-up treatment of any investigational therapy within 6 months before the procedure and/or intent to participate in any other investigational drug or cell therapy study during the 24-month follow-up period of this study; (11) Recipient of prior allogeneic stem cell/progenitor cell therapy; (12) Undergoing a simultaneous procedure to the opposite knee; (13) Injury was work related and covered by workers compensation; (14) A medical condition, serious intercurrent illness, or extenuating circumstance that, in the opinion of the investigator, would preclude participation in the study or potentially decrease survival or interfere with ambulation or rehabilitation (e.g., histories of transient ischemic attack, stroke, uncontrolled diabetes, or liver disease); (15) Presence of ≥20% anti-human leukocyte antigen (HLA) antibody titres and/or having antibody specificities to donor HLAs; (16) History or current evidence of alcohol or drug abuse or was a recreational user of illicit drugs or prescription medications; (17) Significant damage to the collateral or posterior ligaments of the knee; (18) Meniscal injury requiring more than a 1/3 resection or more than a single suture to reconstruction or a reconstruction that would alter the usual ACL rehabilitation; (19) History of prior surgery to the study knee joint; (20) History of malignancy (excluding basal cell carcinoma that has been successfully excised); (21) Chondral lesions noted at time of surgical reconstruction greater than grade 1a on any surfaces; (22) Intra-articular steroid or corticosteroid or HA injections in preceding 3 months to the affected joint; (23) Diffuse synovitis at time of surgery for the ACL reconstruction; (24) Indwelling metal of any description which precluded MRI examination such as, but not limited to, indwelling pacemaker, cerebral aneurysm clips, or electrical indwelling device such as bone stimulator or anything that would preclude patient from undergoing screening MRI; (25) Not willing to return for required follow-up visits or there was a clear demonstration of likely poor compliance; (26) Any other medical condition that, in the judgment of the Principal Investigator/Investigator, would prohibit the patient from participating in the study; (27) Patient was legally or mentally incapacitated; (28) Prisoners or patients who were involuntarily incarcerated; (29) Patients who were compulsorily detained for treatment of a psychiatric disorder.

### Randomisation and intervention

Eligible participants were randomly assigned in a 2:1 fashion to receive intra-articular injection of either 75 million allogeneic MPCs suspended in HA (*n* = 11; the MPC + HA group) or HA alone (*n* = 6). Each consenting patient was assigned a three-digit patient identification number in consecutive, ascending, and chronological order. The unblinded designee not involved in study assessments consulted a central master randomisation list prepared by the project statistician, assigned a patient randomisation number, and allocated treatment in chronological ascending order. The intra-articular injection was performed under ultrasound guidance (to ensure that the interventions were injected into the joint space) by a blinded radiologist with experience in intra-articular injections. After injection, the knee was gently flexed five times and the patients then remained in a supine position for 2 h. Vital signs and adverse events were recorded every 30 min. Concomitant medications were recorded along with the pain score on a visual analogue scale for the knee when resting, moving, and bending. After 2 h, if the patient experienced no untoward adverse events he/she could return home with written follow-up instructions. All patients followed a standard of care rehabilitation programme for ACL reconstruction.

**Fig. 1:**
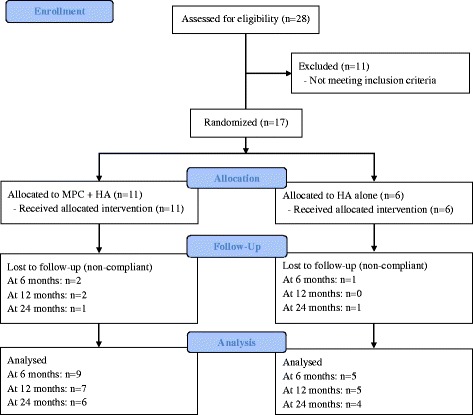
Study flowchart. *HA* hyaluronan, *MPC* mesenchymal precursor cell

The study flowchart is shown in Fig. 1. Nine MPCs + HA and 5 HA-alone patients completed the visit week 26 post-injection; 7 MPCs + HA and 5 HA-alone patients completed the visit week 52 post-injection; and 6 MPCs + HA and 4 HA-alone patients completed the final visit week 104 post-injection.

### Investigational agents

The STRO-3^+^ MPCs used for this study were provided by Mesoblast Ltd. (Level 38, 55 Collins Street, Melbourne, Victoria 3000, Australia). They were derived from adult bone marrow aspirates of young unrelated donors who were not human leukocyte antigen (HLA) matched, purified by stromal immune-selection, culture expanded, and cryopreserved using the company’s proprietary good manufacturing practice (GMP) procedures and product release criteria approved by the TGA. Allogeneic use of the MPCs for this study did not require donor recipient matching or recipient immunosuppression. The MPCs (150 million cells in 4 mL cryopreservent) were supplied for the study in cryobags maintained in the vapour phase of liquid nitrogen at –140 to –196 degrees C. Immediately, prior to application, the cryobags and contents were thawed and 2 mL (75 million MPCs) drawn up into a syringe and mixed using a three-way tap, with 2 mL HA (sodium hyaluronate 10 mg/mL as Euflexxa®, Ferring Pharmaceuticals Inc., Parsippany, NJ, USA) from a pre-filled syringe.

### Safety and tolerance

Adverse events were assessed via a telephone call on the day after the knee injection. Safety measures, including physical examination, vital signs, knee physical assessment, adverse events, laboratory parameters, and documentation of concomitant medications or therapies, were performed at 5 days, 28 days, and 8, 12, 26, 36, 52, 78, and 104 weeks following intra-articular injection. Laboratory blood haematological and biochemical tests included non-fasting chemistry (albumin, alkaline phosphatase, alanine aminotransferase, aspartate aminotransferase, blood urea nitrogen, creatinine, chloride, direct bilirubin, gamma-glutamyl transferase, glucose, lactic dehydrogenase, sodium, potassium, phosphorous, total bilirubin, total calcium, carbon dioxide, total cholesterol, total protein, and uric acid), erythrocyte sedimentation rate and C-reactive protein, complete blood count, HIV, and hepatitis B and hepatitis C viral testing. Because of the human origin of the MPCs and their culture expansion that included exposure to fetal bovine serum, the generation and persistence of anti-HLA panel reactive antibodies (PRA), anti-murine, and anti-bovine antibodies were tested. Immunogenicity was evaluated by anti-HLA PRA against class I and II HLAs measured by flow cytometry.

Additional safety monitoring was implemented by establishing an independent Safety Review Committee. The Safety Review Committee was an independent multidisciplinary group (independent of both the sponsor and contract research organisation) consisting of one biostatistician and three physicians with orthopaedic/rheumatologic expertise who collectively had experience in the management of patients and the conduct and monitoring of randomised clinical trials.

### Knee pain, function, and quality of life

Pain, function, and quality of life were assessed using Knee Injury and OA Outcome Score (KOOS) [33] and SF-36v2 [34] at the time of screening, randomisation (baseline), and 6, 12, 18, and 24 months after intra-articular injection of the test substances. Patient responses to intra-articular treatment using these clinical instruments were determined by calculating the change from their baseline score at 6, 12, 18, and 24 months. Thus, a positive value indicated an improvement and a negative value indicated a worsening.

### Knee x-ray and joint space width measurement

Knee x-ray was performed using a standardised technique, with views including weight-bearing anteroposterior and lateral, skyline and Rosenberg. Participants underwent plain x-rays at screening (baseline) and 6, 12, 18, and 24 months after intra-articular injection and signs of OA were assessed using the Kellgren and Lawrence five-point grading system [35]. Joint space width was measured on the Rosenberg view [36] in millimetres using electronic callipers on a workstation (Inteleviewer, Intelerad, Montreal, Canada). Change in joint space width was calculated as a follow-up measure of change from the baseline, such that a positive value indicated an increase in joint space width and a negative value indicated joint space narrowing.

### MRI acquisition and knee structure measurements

Knee structures were assessed from MRIs performed at screening (baseline), and 6, 12, and 24 months after injection. Knees were imaged in the sagittal plane on a 1.5-T whole body magnetic resonance unit using a commercial transmit-receive extremity coil (Signa HDxt; GE Medical Systems, USA). The following sequence parameters were used: a T_1_-weighted fat suppressed 3D gradient recall acquisition in the steady state; flip angle 40 degrees; repetition time 23 ms; echo time 6.3 ms; field of view 16 cm; 64 partitions; 512 × 512 matrix; one acquisition. Sagittal images were obtained at a partition thickness of 1.5 mm and an in-plane resolution of 0.31 × 0.31 mm. In addition, a coronal proton density fat-saturated acquisition, repetition time 5000 ms, echo time 57 ms, slice thickness 3.5 mm, 1 excitation, a field of view of 16 cm, and a matrix of 512 × 512 pixels was also obtained. Each MRI measurement was performed by a trained observer with an independent cross-check performed by a second trained observer, both blinded to the characteristics of participants, the group allocation of the participants, and the sequence of MRIs. Knee joint articular cartilage pathology was graded using the Osteoarthritis Research Society International grading system [37].

Tibial cartilage volume was determined by image processing on an independent workstation using the software Osiris (Digital Imaging Unit, University Hospital of Geneva, Switzerland). The volumes of medial and lateral tibial cartilage plates were isolated from the total volume by manually drawing disarticulation contours around the cartilage boundaries on each section. The volume of the particular cartilage plate was determined by summing the pertinent voxels within the resultant binary volume. The coefficients of variation (CVs) for cartilage volume measures were 3.4% for medial tibia and 2.0% for lateral tibia [38]. Annual change in cartilage volume was calculated as: (follow-up cartilage volume – baseline cartilage volume) divided by the period of time between MRI scans. Annual percentage change was obtained by dividing annual change by baseline cartilage volume, expressed as a percentage. Therefore, a positive value indicated an increase in cartilage volume and a negative value indicated cartilage volume loss.

Medial and lateral cross-sectional areas of the tibial plateau were determined by creating an isotropic volume from the input images which were reformatted in the axial plane, using the software program Osiris. Areas were directly measured from these axial images as previously described [39]. CVs for the medial and lateral tibial plateau area were 2.3% and 2.4%, respectively [38]. Annual change in bone area was calculated as: (follow-up bone area – baseline bone area) divided by the period of time between MRI scans. Annual percentage change was obtained by dividing annual change by baseline bone area, expressed as a percentage. Thus, a positive value indicated bone expansion and a negative value indicated reduced bone area.

### Anthropometric measures

At screening, each participant had their height and weight measured and body mass index calculated.

### Sample size calculation

The primary aim of this study was to assess the safety and tolerance, and the secondary aim was to explore the preliminary effect of the investigational agents on clinical and knee structural outcomes in patients following ACL reconstruction. The sample size of 24 in this exploratory study was not based on attaining sufficient statistical power for tests of within- or between-group comparisons. There was no formal rule to stop the study based on the number of adverse events within a group. It was possible to estimate the least chance of an adverse event in an individual subject so that one or more groups of 8 subjects were likely to have one or more events.

### Statistical analyses

Descriptive statistics for characteristics of the study participants were tabulated. Continuous variables were assessed for normality. Independent samples *t* tests were used to compare means, and Chi-squared tests used to compare nominal characteristics between the two groups. Least squares analysis was used to compare the change in joint space width from baseline by time point between the two groups. There was no adjustment for multiple comparison performed in this study. A *p* value less than 0.05 (two-tailed) was regarded as statistically significant. All analyses were performed using IBM SPSS version 23.

## Results

### Baseline characteristics of study participants

The baseline characteristics of the study participants are shown in Table 2. There were no significant differences between the two groups in terms of age, gender, body mass index, tibial cartilage volume, tibial bone area, joint space width, tibial cartilage volume, or bone area. Apart from the KOOS activities of daily living (ADL) scores for the HA injected individuals, both groups exhibited KOOS baseline scores that were below the cut-off scores for individuals with physical problems associated with injured knees [7]. However, participants in the MPC + HA group reported worse KOOS pain (*p* = 0.02), symptoms (*p* < 0.001), ADL score (*p* = 0.047), and SF-36v2 bodily pain (*p* = 0.01) score than the HA-alone group at baseline. No significant differences in baseline characteristics were observed between completers and non-completers at 6, 12, and 24 months of follow-up as shown in Fig. 1 (Additional files 1, 2 and 3: Tables S1–S3).

**Table 2:** Baseline characteristics of study participants

	MPC + HA (*n* = 11)	HA alone (*n* = 6)	p*
Age, years	26.0 (3.6)	26.9 (10.3)	0.85
Females, number (%)	3 (27)	2 (33)	0.79
Body mass index, kg/m^2^	25.1 (3.1)	25.1 (4.6)	1.00
Interval between ACL injury and reconstruction, days	76.5 (54.3)	61.7 (34.8)	0.56
Interval between ACL reconstruction and intra-articular injection, days	48.5 (14.0)	47.8 (8.3)	0.92
KOOS			
Pain	69.1 (11.7)	84.2 (11.9)	0.02
Symptoms	59.7 (10.8)	83.9 (11.7)	<0.001
Activities of daily living	82.1 (11.0)	92.8 (6.4)	0.047
Sport and recreation function	29.7 (17.6)	40.0 (14.1)	0.47
Knee-related quality of life	40.9 (19.8)	50.0 (16.8)	0.36
SF-36 physical component score	41.3 (4.7)	45.9 (5.8)	0.11
Physical functioning	41.0 (6.2)	43.7 (1.7)	0.31
Role limitation: physical	40.0 (7.0)	42.6 (8.1)	0.51
Bodily pain	42.3 (8.6)	53.5 (5.4)	0.01
General health perception	55.2 (6.6)	55.4 (7.9)	0.95
Joint space width, mm			
Medial tibiofemoral compartment	4.36 (0.55)	4.97 (0.63)	0.06
Lateral tibiofemoral compartment	5.38 (0.62)	5.67 (0.52)	0.36
Knee structure measured from MRI			
Medial tibial cartilage volume, mm^3^	2456 (397)	2627 (476)	0.44
Lateral tibial cartilage volume, mm^3^	3291 (401)	3548 (847)	0.51
Medial tibial plateau bone area, mm^2^	2299 (337)	2187 (182)	0.46
Lateral tibial plateau bone area, mm^2^	1472 (202)	1415 (193)	0.58

Data are reported as mean (SD) or number (%)

*For difference between two groups using independent samples *t* test or chi-squared test where appropriate

*ACL* anterior cruciate ligament, *KOOS* Knee Injury and Osteoarthritis Outcome Score, *MRI* magnetic resonance imaging

### Adverse events

The incidence of adverse events was graded according to the National Cancer Institute Common Terminology Criteria for Adverse Events (NCICTCAE) and is summarised in Table 3. No participants experienced treatment-related or treatment-emergent serious adverse events that resulted in death, treatment discontinuation, or study termination over the course of the study.

**Table 3:** Incidence of adverse events

	MPC + HA (*n* = 11)	HA alone (*n* = 6)
Total number of adverse events	94	39
Non-serious, *n* (%)	92 (97.9)	39 (100.0)
Serious, *n* (%)	2 (2.1)^#^	0 (0.0)
Adverse events by system organ class*, *n* (grade)		
Musculoskeletal and connective tissue disorders	11 (2)	6 (1)
General disorders and administration site conditions	3 (2)	1 (1)
Injury, poisoning, and procedural complications	2 (1)	2 (1)
Infections and infestations	1 (1)	1 (1)
Respiratory, thoracic, and mediastinal disorders	1 (1)	0
Immune system disorders	2 (2)	1 (2)

* Graded according to the National Cancer Institute Common Terminology Criteria for Adverse Events (NCICTCAE)

^#^ Non-treatment related

In the MPC + HA group, increases in class I PRA >10% were observed in 5 out of 6 patients at week 4, in 5 out of 7 patients at week 36, in 2 out of 7 patients at week 78, and in 1 out of 6 patients at week 104. In the HA-alone group, only 1 out of 4 patients had increases in class I PRA >10% at week 4, and this level was maintained through to week 104. None of the study participants exhibited clinically significant abnormalities in haematology or blood chemistry laboratory results over the course of the study. No major changes in vital signs were reported following injection of the test substances, nor were there notable changes from baseline in the physical examination data. No ectopic tissue formation was noted from the blinded MRI evaluations.

### Serious adverse events

Two serious adverse events, fracture of the humerus and infective bursitis, were reported for patients in the MPC + HA group after week 52. These were not considered to be treatment related.

### Effect of MPCs on knee pain, function, and quality of life over 24 months

**Fig. 2:**
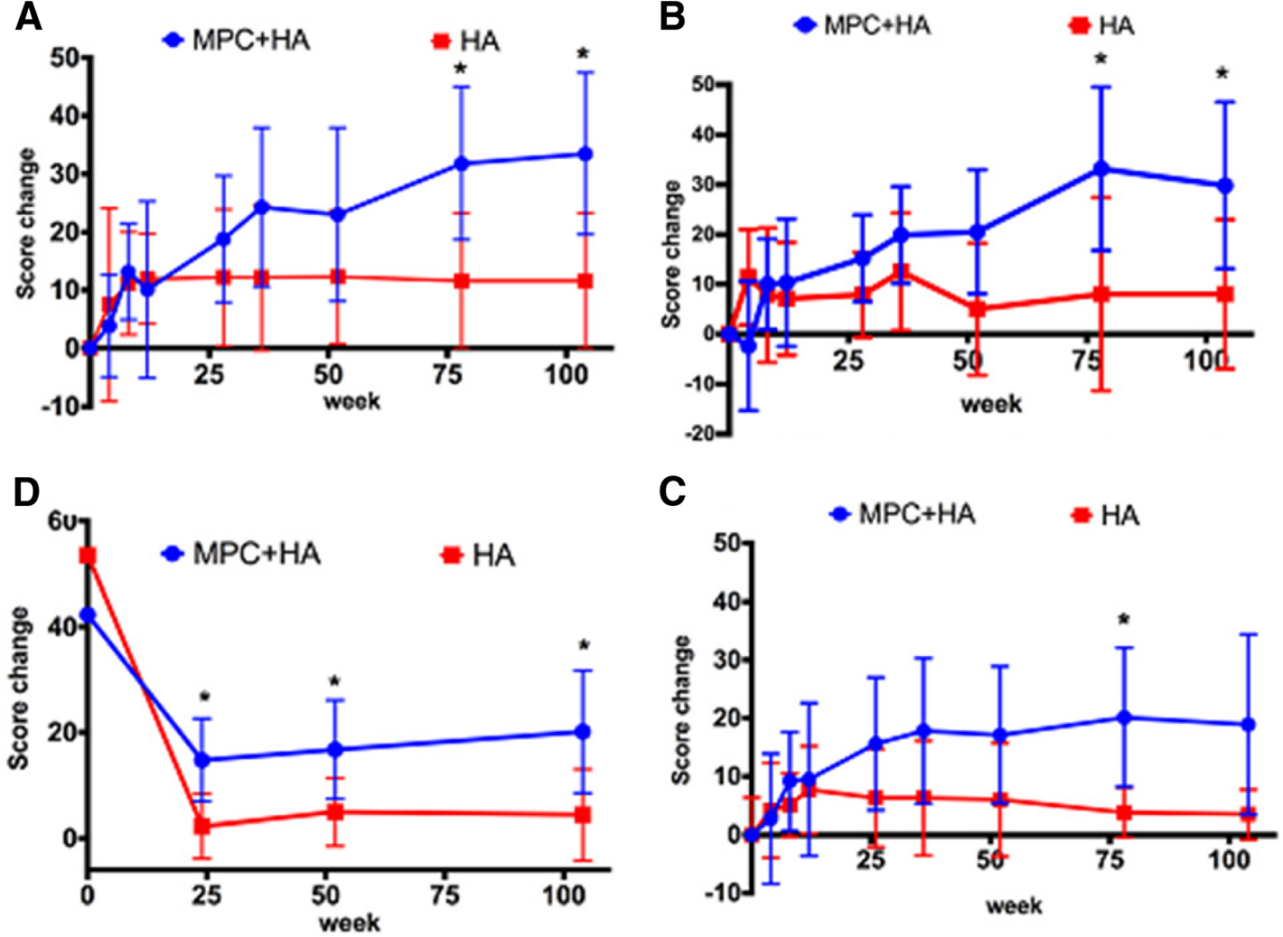
Change from baseline in **a–c** Knee Injury and Osteoarthritis Outcome Score (*KOOS*) (pain scores (**a**), symptom scores (**b**), and activities of daily living (*ADL*) scores (**c**)), and **d** SF-36 physical component scores over 24 months (mean and 95% confidence interval shown). *p <﻿ 0.05﻿ *HA* hyaluronan, *MPC* mesenchymal precursor cell

There was an improvement from baseline in both groups for all the KOOS dimensions over 24 months (Fig. 2, Additional file 4: Table S4). However, significant differences between treatment groups were observed for KOOS symptoms and pain at 18 months (both *p* = 0.03) and 24 months (*p* = 0.04 and 0.02, respectively), and for ADL at 18 months (*p* = 0.04), all in favour of patients who were injected with the MPC + HA preparation (Fig. 2, Additional file 4: Table S4). There was also an improvement over 24 months in both groups for all SF-36 physical components except for general health perception (Fig. 2, Additional file 5: Table S5). However, statistically significant differences between treatments were only observed for bodily pain at 6, 12, and 24 months (*p* = 0.02, 0.05, and 0.03, respectively), where injection with the MPH + HA preparation was found to be more beneficial than HA alone (Fig. 2, Additional file 5: Table S5).

### Effect of MPCs on tibial cartilage volume change over 24 months

There was no significant difference in annual change in medial or lateral tibial cartilage volume between the MPC + HA and HA-alone groups over 6, 12, and 24 months (Table 4). However, the MPC + HA group tended to have a reduced rate of medial tibial cartilage volume loss compared with the HA-alone group over the first 6 months of the study (0.7% vs –4.0%, *p* = 0.10) (Table 4).

**Table 4:** Annual percentage change from baseline in tibial cartilage volume over 24 months

	MPC + HA	HA alone	p*
Medial tibia			
6 months	0.7 (5.9)	–4.0 (3.9)	0.10
12 months	0.3 (6.3)	–2.4 (3.1)	0.36
24 months	–1.4 (4.2)	–3.3 (5.3)	0.54
Lateral tibia			
6 months	–1.4 (5.3)	–2.7 (4.4)	0.65
12 months	–4.7 (3.4)	–2.6 (2.5)	0.25
24 months	–3.7 (3.4)	–0.8 (3.5)	0.22

Data are reported as mean (SD)

* For difference between two groups using independent samples *t* test

*HA* hyaluronan, *MPC* mesenchymal precursor cell

### Effect of MPCs on tibial bone expansion over 24 months

The rate of total tibial bone expansion in the MPC + HA group showed a significantly decrease compared with the HA-alone group over the first 6 months post-treatment (0.5% vs 4.0%, *p* = 0.02) (Table 5). The trend was maintained over the subsequent 12 and 24 months (both *p* = 0.09).

**Table 5:** Annual percentage tibial bone expansion from baseline over 24 months

	MPC + HA	HA alone	p*
Medial tibial plateau			
6 months	0.8 (3.9)	4.2 (3.4)	0.13
12 months	–1.9 (4.0)	1.5 (3.6)	0.17
24 months	0.1 (1.7)	0.8 (0.9)	0.43
Lateral tibial plateau			
6 months	0.4 (4.2)	3.6 (3.6)	0.17
12 months	–0.2 (4.0)	1.9 (2.8)	0.35
24 months	–1.8 (2.6)	1.2 (3.5)	0.16
Total tibial plateau			
6 months	0.5 (2.4)	4.0 (2.3)	0.02
12 months	–1.2 (2.8)	1.7 (2.0)	0.09
24 months	–0.7 (1.5)	1.0 (1.1)	0.09

Data are reported as mean (SD)

* For difference between two groups using independent samples *t* test

*HA* hyaluronan, *MPC* mesenchymal precursor cell

### Effect of MPCs on tibiofemoral joint space width over 24 months

**Fig. 3:**
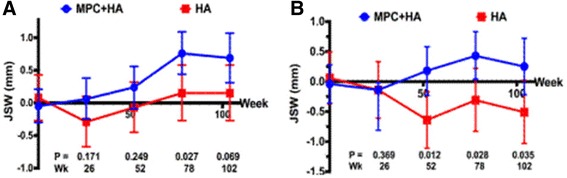
Change from baseline in tibiofemoral joint space width over 24 months (﻿medial joint space width (**a**) and lateral joint space width (**b**)) (mean and 95% confidence interval shown). *HA* hyaluronan, *MPC* mesenchymal precursor cell

Radiological assessments of the mean joint space width of patients when expressed as individual changes from baseline revealed a greater increase in the MPC + HA group than the HA-alone group at 12, 18, and 24 months post-administration (Fig. 3, Additional file 6: Table S6). In the medial compartment, injection with MPC + HA resulted in a greater joint space width increase compared with the HA-alone group at 18 months (*p* = 0.03) and 24 months (*p* = 0.07) (Fig. 3, Additional file 6: Table S6). In the lateral compartment, joint space width increased relative to baseline in the MPC + HA group but declined in the HA-alone group. This resulted in significant differences between treatment at 12 months (*p* = 0.01), 18 months (*p* = 0.03), and 24 months (*p* = 0.04) (Fig. 3, Additional file 6: Table S6).

## Discussion

This study is the first to evaluate the therapeutic effects of a single intra-articular injection of allogeneic immuno-selected MPCs on the long-term (2 years) outcomes of knee joint symptoms, cartilage, and subchondral bone changes post-ACL reconstruction. The study showed that intra-articular administration of allogeneic MPCs was safe and well tolerated over the 24-month study period following a single injection. Moreover, using validated clinical instruments under blinded conditions, there was evidence for improvements in pain, function, and quality of daily life, as well as favourable effects on knee joint structural changes over 24 months, supporting further investigation of MPCs as potential disease-modifying agents in post-traumatic knee OA.

In terms of both safety and efficacy our findings were consistent with previous studies using culture-expanded autologous bone marrow-derived MSC administered via the intra-articular route for the management of OA and a variety of other joint problems [16, 19, 40–44]. Over our 2-year study no cell-related serious adverse events or abnormalities in laboratory parameters or clinical signs were observed.

Although the patient group enrolled in our study were young adults with a recent ACL reconstruction who exhibited no radiographic signs of OA, they reported persistent joint pain at study entry with the MPC + HA group exhibiting lower (worse) KOOS pain, symptom, and ADL scores than the HA group at baseline. Over the course of the study both MPC + HA- and HA-injected groups showed symptomatic improvement; however, the MPC + HA-treated group exhibited greater changes from baseline.

Although the primary aim of the study was to examine the safety and tolerability of the test substances, it also demonstrated favourable effects of MPCs on knee structural outcomes relative to the HA-alone group. We found that tibial bone expansion was halted in the MPC + HA arm (0.5 ± 2.4%), while there was a significant bone expansion in the HA alone group (4.0 ± 2.3%) over 6 months, with a non-statistically significant trend observed at 12 and 24 months. Although we did not observe a statistically significant effect of MPCs on knee cartilage, there was a trend for slowing in cartilage volume loss in the MPC + HA group (0.7 ± 5.9%) compared to the HA-only group (–4.0 ± 3.9%) over 6 months. This was supported by the finding of reduced joint space narrowing in the MPC + HA group compared to the HA-only group. Recent studies have shown that the earliest changes at the knee post-trauma are seen at the subchondral bone [45]. Hunter and colleagues showed that both ACL injury and ACL reconstruction were associated with significant flattening of articulating bone curvature over 5 years [45]. We showed that there was tibial bone expansion at 2 and 4 years post-meniscectomy which predated changes in cartilage volume [46]. Subchondral bone and cartilage are intimately integrated, since the avascular cartilage relies on the integrity of vascularized subchondral bone to remain functional. A greater tibial plateau bone area has been shown to be associated with the classical radiographic hallmarks of OA (osteophyte and joint space narrowing) [47] and cartilage defects [48]. Taken together, these preliminary results suggest that the administration of MPCs in post-ACL reconstruction could slow the rate of disease progression, thereby delaying the onset of post-traumatic OA in later years.

The mechanisms of action responsible for the beneficial effect of MPCs on patient-reported clinical outcomes and radiograph- and MRI-derived structural outcomes are presently unresolved. However, MPCs are known to exhibit anti-inflammatory and immunomodulatory activities [11–14] which could suppress the injury-induced production of inflammatory mediators within joint tissues that are responsible for the clinical symptoms and breakdown of joint connective tissues [2]. The MPCs have surface receptors for interleukin (IL)-6, tumour necrosis factor (TNF), IL-1, and IL-17, among others, that bind these cytokines when induced at sites of tissue injury and inflammation. The receptor engagement results in secretion by the MPCs of prostaglandin E2 and indoleamine 2,3-dioxygenase which can polarise monocytes to an anti-inflammatory M2 phenotype and T helper 17 cells to FoxP3 T regulatory cells [14, 49]. This pathway results in increased IL-10 levels and reduction in the very inflammatory cytokines that initiated the response [30, 31, 50]. Moreover, co-culture of human synovial and cartilage explants exposed to conditioned media from MSC pre-treated with TNF-alpha showed reduced expression of IL-beta and matrix metalloproteinases and upregulation of the cytokine signalling suppressor 1 gene while, in cartilage, upregulation of IL-1 receptor antagonist and downregulation of the proteoglycan degrading proteinase ADAMTS-5 was observed [51]. MPCs injected intravenously to sheep with collagen-induced joint inflammation was demonstrated to decrease cartilage erosions, synovial stromal cell activation, angiogenesis, and plasma levels of activin A and IL-17A, confirming that systemic administration of these cells can modulate both local joint and systemic inflammation [32].

The present study has limitations. It is a pilot phase Ib/IIa randomised controlled trial with the primary aim being to determine the safety and tolerability of a single intra-articular injection of allogeneic MPCs. The small sample size has limited the power of our study to show significant results for some structural and clinical outcomes such as cartilage volume loss and ADL score. However, it has demonstrated consistent findings between clinical symptoms and knee structural changes. In terms of joint structural effects, we have observed a more pronounced effect of MPCs on subchondral bone remodelling accompanied by a preservative effect on articular cartilage which follows a biologically plausible sequence of effect, particularly after ACL reconstruction. Although there was a higher rate of loss to follow-up (17.6% at 6 months, 29.4% at 12 months, and 41.2% at 24 months), there were no significant differences between the completers and non-completers in terms of baseline characteristics of age, gender, body mass index, tibial cartilage volume, tibial bone area, or joint space width. The strengths of our study included the objective assessment of changes in cartilage volume and bone area over 24 months at four time points which were measured from MRI using validated methods. Both MRI outcome measures have been shown to play a role in the pathogenesis of knee OA [48, 52]. Furthermore, each measurement was independently performed by a trained observer who was blinded to the participant characteristics, the group allocation of the participants, and the sequence of MRIs, with high intra-observer reproducibility.

## Conclusions

Intra-articular administration of a single injection of allogeneic immuno-selected MPCs following ACL reconstruction was shown to be safe and well tolerated in this 24-month study. There was also preliminary evidence to support the efficacy of MPCs in improving both symptoms and structural outcomes. These findings suggest that MPCs may modulate some of the pathological processes responsible for the onset and progression of post-traumatic OA and warrant further investigation of their potential as a disease-modifying agent for the treatment of early joint injuries.

## Additional files

Additional file 1: **Table S1.** Baseline characteristics of completers and non-completers at 6 months. (DOC 32 kb)

Additional file 2: **Table S2.** Baseline characteristics of completers and non-completers at 12 months. (DOC 32 kb)

Additional file 3: **Table S3.** Baseline characteristics of completers and non-completers at 24 months. (DOC 32 kb)

Additional file 4: **Table S4.** Change from baseline in KOOS scores over 24 months. (DOC 46 kb)

Additional file 5: **Table S5.** Change from baseline in SF-36 physical component scores over 24 months. (DOC 42 kb)

Additional file 6 **Table S6.** Radiologically determined change in joint space width from baseline over 24 months. (DOC 36 kb)

## Abbreviations

ACL: Anterior cruciate ligament
ADL: Activities of daily living
CFU-F: Colony-forming units-fibroblast
cGCP: Criteria compliant with principles of good clinical practice
CV: Coefficient of variation
GMP: Good manufacturing practice
HA: Hyaluronan
HLA: Human leukocyte antigen
IL: Interleukin
KOOS: Knee Injury and Osteoarthritis Outcome Score
MPC: Mesenchymal precursor cell
MRI: Magnetic resonance imaging
MSC: Mesenchymal stem cells
OA: Osteoarthritis
PRA: Panel reactive antibodies
TGA: Therapeutic Goods Administration
TNF: Tumour necrosis factor
