# Supplementary material for: Safety, tolerability, clinical, and joint structural outcomes of a single intra-articular injection of allogeneic mesenchymal precursor cells in patients following anterior cruciate ligament reconstruction: a controlled double-blind randomised trial
Source: Arthritis Res Ther. 2017 Aug 2;19:180. doi: 10.1186/s13075-017-1391-0 (PMC5541727; doi:10.1186/s13075-017-1391-0)
Supplement: Supplementary file 1 — Table S1. Baseline characteristics of completers and non-completers at 6 months. (DOC 32 kb) [file 13075_2017_1391_MOESM1_ESM.doc]

**Additional file 1: Table S**1. Baseline characteristics of completers and non-completers at 6 month

|  | Completers  N = 14 | Non-completers  N = 3 | P * |
| --- | --- | --- | --- |
| Age, years | 27.0 (6.8) | 23.3 (3.8) | 0.39 |
| Females, number (%) | 4 (29) | 1 (33) | 1.00 |
| Body mass index, kg/m^2^ | 25.3 (3.6) | 24.3 (3.5) | 0.68 |
| Medial tibial cartilage volume, mm^3^ | 2496 (399) | 2611 (603) | 0.68 |
| Lateral tibial cartilage volume, mm^3^ | 3348 (580) | 3538 (705) | 0.63 |
| Medial tibial plateau bone area, mm^2^ | 2295 (300) | 2095 (203) | 0.29 |
| Lateral tibial plateau bone area, mm^2^ | 1457 (209) | 1428 (136) | 0.82 |

Data were reported as mean (SD) or number (%)

*for difference between 2 groups using independent samples t test or chi-squared test where appropriate
