# Supplementary material for: Safety, tolerability, clinical, and joint structural outcomes of a single intra-articular injection of allogeneic mesenchymal precursor cells in patients following anterior cruciate ligament reconstruction: a controlled double-blind randomised trial
Source: Arthritis Res Ther. 2017 Aug 2;19:180. doi: 10.1186/s13075-017-1391-0 (PMC5541727; doi:10.1186/s13075-017-1391-0)
Supplement: Supplementary file 2 — Table S2. Baseline characteristics of completers and non-completers at 12 months. (DOC 32 kb) [file 13075_2017_1391_MOESM2_ESM.doc]

**Additional file 2: Table S**2. Baseline characteristics of completers and non-completers at 12 month

|  | Completers  N = 12 | Non-completers  N = 5 | P * |
| --- | --- | --- | --- |
| Age, years | 27.0 (7.2) | 24.6 (4.4) | 0.50 |
| Females, number (%) | 4 (33) | 1 (20) | 1.00 |
| Body mass index, kg/m^2^ | 25.8 (3.4) | 23.5 (3.6) | 0.25 |
| Medial tibial cartilage volume, mm^3^ | 2464 (340) | 2643 (601) | 0.56 |
| Lateral tibial cartilage volume, mm^3^ | 3410 (590) | 3314 (632) | 0.77 |
| Medial tibial plateau bone area, mm^2^ | 2273 (294) | 2226 (313) | 0.77 |
| Lateral tibial plateau bone area, mm^2^ | 1479 (219) | 1387 (113) | 0.40 |

Data were reported as mean (SD) or number (%)

*for difference between 2 groups using independent samples t test or chi-squared test where appropriate
