# Supplementary material for: Safety, tolerability, clinical, and joint structural outcomes of a single intra-articular injection of allogeneic mesenchymal precursor cells in patients following anterior cruciate ligament reconstruction: a controlled double-blind randomised trial
Source: Arthritis Res Ther. 2017 Aug 2;19:180. doi: 10.1186/s13075-017-1391-0 (PMC5541727; doi:10.1186/s13075-017-1391-0)
Supplement: Supplementary file 3 — Table S3. Baseline characteristics of completers and non-completers at 24 months. (DOC 32 kb) [file 13075_2017_1391_MOESM3_ESM.doc]

**Additional file 3: Table S**3. Baseline characteristics of completers and non-completers at 24 month

|  | Completers  N = 10 | Non-completers  N = 7 | P * |
| --- | --- | --- | --- |
| Age, years | 27.2 (7.9) | 25.0 (3.9) | 0.50 |
| Females, number (%) | 4 (40) | 1 (14) | 0.34 |
| Body mass index, kg/m^2^ | 24.8 (2.6) | 25.6 (4.8) | 0.65 |
| Medial tibial cartilage volume, mm^3^ | 2374 (295) | 2720 (508) | 0.14 |
| Lateral tibial cartilage volume, mm^3^ | 3302 (582) | 3495 (613) | 0.52 |
| Medial tibial plateau bone area, mm^2^ | 2202 (248) | 2342 (346) | 0.34 |
| Lateral tibial plateau bone area, mm^2^ | 1442 (221) | 1465 (165) | 0.82 |

Data were reported as mean (SD) or number (%)

*for difference between 2 groups using independent samples t test or chi-squared test where appropriate
