# Supplementary material for: Safety, tolerability, clinical, and joint structural outcomes of a single intra-articular injection of allogeneic mesenchymal precursor cells in patients following anterior cruciate ligament reconstruction: a controlled double-blind randomised trial
Source: Arthritis Res Ther. 2017 Aug 2;19:180. doi: 10.1186/s13075-017-1391-0 (PMC5541727; doi:10.1186/s13075-017-1391-0)
Supplement: Supplementary file 4 — Table S4. Change from baseline in KOOS scores over 24 months. (DOC 46 kb) [file 13075_2017_1391_MOESM4_ESM.doc]

Additional file 4: Table S4: Change from baseline in KOOS scores over 24 months

| KOOS dimension | | MPC + HA | HA alone | P* |
| --- | --- | --- | --- | --- |
| **Symptoms** |  | |  |  |
| 6 months | 15.2 (6.5, 23.9) | | 7.9 (-0.7, 16.4) | 0.19 |
| 12 months | 20.5 (8.1, 33.0) | | 5.0 (-8.2, 18.2) | 0.07 |
| 18 months | 33.2 (16.8, 49.5) | | 8.0 (-11.3, 27.4) | 0.03 |
| 24 months | 29.8 (13.1, 46.5) | | 8.0 (-6.9, 23.0) | 0.04 |
| **Pain** |  | |  |  |
| 6 months | 18.8 (7.9, 29.7) | | 12.2 (0.4, 23.9) | 0.35 |
| 12 months | 23.0 (8.1, 38.0) | | 12.3 (0.8, 23.8) | 0.24 |
| 18 months | 31.8 (18.7, 45.0) | | 11.6 (0.0, 23.3) | 0.03 |
| 24 months | 33.5 (19.6, 47.4) | | 11.6 (0.0, 23.3) | 0.02 |
| **Activities of daily living** |  | |  |  |
| 6 months | 15.6 (4.2, 27.0) | | 6.3 (-2.1, 14.7) | 0.19 |
| 12 months | 17.1 (5.4, 28.9) | | 6.0 (-3.7, 15.7) | 0.14 |
| 18 months | 20.1 (8.2, 32.1) | | 3.8 (-0.4, 8.1) | 0.04 |
| 24 months | 18.9 (3.4, 34.4) | | 3.5 (-0.8, 7.7) | 0.08 |
| **Sport and recreation function** |  | |  |  |
| 6 months | 35.3 (6.6, 64.0) | | 32.5 (-189.9, 254.9) | 0.92 |
| 12 months | 47.8 (15.4, 80.3) | | 47.5 (-238.4, 333.4) | 0.99 |
| 18 months | 59.1 (24.8, 93.4) | | 55.0 (-135.6, 245.6) | 0.89 |
| 24 months | 59.2 (19.3, 99.0) | | 60.0 (-67.1, 187.1) | 0.98 |
| **Knee-related quality of life** |  | |  |  |
| 6 months | 16.4 (-3.3, 36.1) | | 20.0 (4.1, 35.9) | 0.76 |
| 12 months | 29.7 (-1.8, 61.1) | | 30.0 (9.2, 50.8) | 0.99 |
| 18 months | 44.6 (26.2, 63.1) | | 37.5 (19.3, 55.7) | 0.53 |
| 24 months | 46.5 (26.1, 67.0) | | 32.8 (10.8, 54.9) | 0.26 |

Data presented as mean change (95% confidence interval)

*difference between treatment groups using independent samples t-test
