# Supplementary material for: Safety, tolerability, clinical, and joint structural outcomes of a single intra-articular injection of allogeneic mesenchymal precursor cells in patients following anterior cruciate ligament reconstruction: a controlled double-blind randomised trial
Source: Arthritis Res Ther. 2017 Aug 2;19:180. doi: 10.1186/s13075-017-1391-0 (PMC5541727; doi:10.1186/s13075-017-1391-0)
Supplement: Supplementary file 5 — Table S5. Change from baseline in SF-36 physical component scores over 24 months. (DOC 42 kb) [file 13075_2017_1391_MOESM5_ESM.doc]

Additional file 5: Table S5: Change from baseline in SF-36 physical component scores over 24 months

| SF-36v2 components | | MPC + HA | HA alone | P* |
| --- | --- | --- | --- | --- |
| **Physical functioning** |  | |  |  |
| 6 months | 11.6 (6.2, 17.0) | | 7.2 (-1.2, 15.5) | 0.26 |
| 12 months | 13.9 (7.2, 20.7) | | 10.5 (4.4, 16.7) | 0.42 |
| 24 months | 16.5 (8.9, 24.1) | | 13.2 (9.9, 16.4) | 0.40 |
| **Role limitation: physical** |  | |  |  |
| 6 months | 9.5 (2.1, 16.9) | | 10.8 (3.8, 17.8) | 0.78 |
| 12 months | 13.5 (5.8, 21.1) | | 12.7 (-1.4, 26.9) | 0.90 |
| 24 months | 18.0 (9.3, 26.7) | | 14.1 (0.1, 28.1) | 0.50 |
| **Bodily pain** |  | |  |  |
| 6 months | 14.7 (6.9, 22.6) | | 2.2 (-3.9, 8.3) | 0.02 |
| 12 months | 16.7 (7.4, 26.1) | | 4.9 (-1.5, 11.3) | 0.05 |
| 24 months | 20.1 (8.5, 31.7) | | 4.4 (-4.2, 13.0) | 0.03 |
| **General health** |  | |  |  |
| 6 months | -1.2 (-7.8, 5.4) | | 1.4 (-1.2, 4.1) | 0.49 |
| 12 months | -1.7 (-6.8, 3.4) | | -0.7 (-4.4, 3.1) | 0.74 |
| 24 months | -1.7 (-9.1, 5.6) | | 6.4 (-8.5, 21.3) | 0.13 |
| **SF-36 physical component score** |  | |  |  |
| 6 months | 9.0 (1.6, 16.3) | | 6.6 (0.6, 12.6) | 0.60 |
| 12 months | 11.2 (4.1, 18.3) | | 10.3 (3.6, 17.0) | 0.85 |
| 24 months | 15.8 (11.1, 20.4) | | 11.8 (2.8, 20.8) | 0.22 |

Data presented as mean change (95% confidence interval)

*difference between treatment groups using independent samples t-test
