# Supplementary material for: Safety, tolerability, clinical, and joint structural outcomes of a single intra-articular injection of allogeneic mesenchymal precursor cells in patients following anterior cruciate ligament reconstruction: a controlled double-blind randomised trial
Source: Arthritis Res Ther. 2017 Aug 2;19:180. doi: 10.1186/s13075-017-1391-0 (PMC5541727; doi:10.1186/s13075-017-1391-0)
Supplement: Supplementary file 6 — Table S6. Radiologically determined change in joint space width from baseline over 24 months. (DOC 36 kb) [file 13075_2017_1391_MOESM6_ESM.doc]

Additional file 6: Table S6: Radiologically determined change in joint space width from baseline over 24 months

|  | MPC + HA | HA alone | Difference | P* |
| --- | --- | --- | --- | --- |
| **Medial compartment, mm** | |  |  |  |
| 6 months | 0.06 (-0.25, 0.38) | -0.29 (-0.67, 0.10) | 0.35 (-0.16, 0.85) | 0.17 |
| 12 months | 0.24 (-0.09, 0.56) | -0.07 (-0.45, 0.32) | 0.30 (-0.22, 0.82) | 0.25 |
| 18 months | 0.76 (0.44, 1.09) | 0.15 (-0.27, 0.58) | 0.61 (0.07, 1.15) | 0.03 |
| 24 months | 0.69 (0.31, 1.07) | 0.15 (-0.27, 0.58) | 0.54 (-0.04, 1.12) | 0.07 |
| **Lateral compartment, mm** | |  |  |  |
| 6 months | -0.41 (-0.81, -0.02) | -0.14 (-0.61, 0.33) | -0.28 (-0.89, 0.34) | 0.37 |
| 12 months | 0.18 (-0.23, 0.58) | -0.64 (-1.11, -0.17) | 0.81 (0.19, 1.44) | 0.01 |
| 18 months | 0.43 (0.04, 0.83) | -0.31 (-0.83, 0.22) | 0.74 (0.08, 1.39) | 0.03 |
| 24 months | 0.25 (-0.22, 0.72) | -0.51 (-1.03, 0.02) | 0.76 (0.05, 1.46) | 0.04 |

Data were reported as mean change (95% confidence interval)

*difference in the least squares mean between treatment groups using the least squares analysis
